# Response of Sagebrush Steppe Grass Species to AMF Inoculum Sources and Biochar

**DOI:** 10.3390/microorganisms11051113

**Published:** 2023-04-25

**Authors:** David Eduardo Prado-Tarango, Ricardo Mata-González, Matthew Hovland

**Affiliations:** Department of Animal and Rangeland Sciences, Oregon State University, Corvallis, OR 97331, USA

**Keywords:** mycorrhiza, inoculation, rangelands, restoration, invasive species, Artemisia, medusahead

## Abstract

The sagebrush steppe has presented increasing levels of degradation. The addition of arbuscular mycorrhizal fungi (AMF) and biochar have been suggested to restore ecosystems. However, little is known about their effects on sagebrush steppe plants. We tested three sources of AMF inoculum: soil from a disturbed site (Inoculum A), soil from an undisturbed site (Inoculum B), and commercial inoculum (Inoculum C), all with and without biochar, to test if they could mediate growth of *Pseudoroegneria spicata* (native perennial), *Taeniatherum caput-medusae* (early seral; exotic annual) and *Ventenata dubia* (early seral; exotic annual) under greenhouse conditions. We measured AMF colonization and biomass. We hypothesized that the plant species would be differently affected by the inoculum types. The colonization of *T. caput-medusae* and *V. dubia* was greatest when inoculated with Inoculum A (38.8% and 19.6%). In contrast, the colonization of *P. spicata* was greatest with Inoculum B and Inoculum C (32.1% and 32.2). Biochar decreased biomass production but increased colonization with Inoculum A for *P. spicata* and *V. dubia* and with Inoculum C for *T. caput-medusae*. This study reveals the response of early and late seral sagebrush steppe grass species to contrasting sources of AMF and suggests that late seral plant species respond better to late seral inocula.

## 1. Introduction

The loss of perennial plant cover is one of the most conspicuous issues in many rangelands of the world [1,2,3]. This loss is caused by factors such as heavy grazing, climate change, wildfires, and invasive species [2,4,5,6]. In the sagebrush steppe of western North America, the perennial plant cover is constantly being replaced by invasive or early seral plant species [7,8]. Invasive species can be particularly problematic as they can disrupt successional processes, alter wildfire return intervals, and reduce habitat quality [9,10,11,12]. Furthermore, they can suppress perennial plant growth by quickly taking resources [13], which hinders the re-establishment of other more desired perennial plant species.

Natural successional processes can repair a degraded ecosystem, but in arid environments natural succession can take decades or hundreds of years [5,14,15], particularly when disturbance levels (such as invasive species) pass a given threshold [16]. In these instances, a restoration program is recommended to accelerate ecological succession [17]. However, ecological restoration programs often fail [18]. Multiple ecological factors such as competition, drought, herbivory, and invasive species need to be properly addressed for a successful restoration program [9,19,20]. To increase restoration success, a restoration program can incorporate different techniques such as inoculation with arbuscular mycorrhizal fungi (AMF) or biochar.

AMF are root endophytes that associate with a host plant and can improve the nutrient uptake of plants, particularly phosphorus (P) and nitrogen (N), and transfer those nutrients to plants in exchange for photosynthates [21]. AMF can enhance tolerance to drought stress by improving the water balance of drought-stressed plants, influence stomatal conductance, and decrease oxidative damage by enhancing enzymatic and non-enzymatic activities [22]. In addition to drought-stress tolerance, studies have found promising results by using AMF and other biofertilizers to reduce environmental stresses such as salinity and heavy metal accumulations [22,23,24]. Due to this, AMF have been gaining interest and are regularly used as a restoration tool, particularly in forested systems [25,26,27,28]. AMF can be particularly important in ecosystems with a history of disturbance with a degraded local AMF propagule density [26,29]. However, questions remain concerning AMF, regarding such topics as colonization and growth [30,31,32,33]. It is also of concern if the application of AMF inoculum can benefit undesired or invasive plant species [34].

It is known that commercial AMF inocula differ from local native soil inocula because of their AMF composition [30,32,35]; thus, variation in the responses to commercial inocula are expected. Some authors found positive plant growth and colonization levels by applying live field soils as AMF inocula taken from undisturbed or late seral ecosystems [27,36,37,38], while other studies have found little or no difference in plant growth when using live soils from disturbed sites or early seral ecosystems [39]. Thus, understanding the potential differential effects of multiple sources of AMF inoculum can be important to devise successful rangeland restoration programs. This is important as some invasive species are known to benefit from associations with AMF [40,41]. If an invasive species benefits from AMF, then inoculation may not be a proper tool for restoration [34].

Another important tool for restoration is biochar, a soil amendment produced by thermal decomposition of different types of organic materials in low oxygen (pyrolysis) conditions [42,43,44]. The resulting product is a porous, carbon-rich product used to increase soil fertility [44,45,46]. Biochar has been used for years in agriculture to enhance soil productivity, plant growth, and soil carbon storage and to improve soil conditions for mycorrhizae [47,48]. However, some experiments reported negative or neutral results in improving AMF colonization with biochar, while others highlighted the existence of tradeoffs [45,48,49]. In addition, much less is known about the effect of biochar in non-agricultural or rangeland plants. This indicates the need to assess biochar application in rangeland restoration to better understand its effects on plant growth and its potential interactions with AMF.

*Pseudoroegneria spicata* (Pursh) Á. Löve (bluebunch wheatgrass), *Taeniatherum caput-medusae* L. (medusahead), and *Ventenata dubia* (Leers) Coss. (ventenata) are important rangeland grass species in the cold deserts of North America. *P. spicata* is a native perennial bunchgrass that is considered a keystone species in sagebrush steppe ecosystems and one of the most abundant grasses [50]. AMF research on this species is limited [51], and varying levels of AMF colonization were reported [52,53]. *T. caput-medusae* is an exotic annual grass species that has successfully invaded multiple sagebrush steppe systems [5,9,10]. There is a limited understanding of the mycorrhizal associations for this species [54]. *V. dubia* is an exotic annual species that has recently invaded the sagebrush steppe [11,12,55], and, thus, its ecology and specifically its AMF interactions have been poorly studied.

A first step for ecosystem restoration with AMF and biochar is to understand plant–AMF interactions and how biochar might mediate such a response. However, AMF may also benefit plants considered weeds, and, therefore, in some cases it can lead to plant invasions [56]. Furthermore, the inoculum source needs to be carefully considered because of its different components and effects [30]. We conducted a greenhouse study to evaluate the growth of *P. spicata*, *T. caput-medusae*, and *V. dubia* to biochar and three different sources of AMF inoculum: (1) soil from a disturbed site (historically plowed, reseeded, and grazed), (2) soil from an undisturbed site, and (3) a commercial AMF inoculum. We hypothesized that (1) each inoculum sources would have differential effects (colonization and biomass production) on each plant species (late seral vs. early seral species) and that (2) biochar would increase root colonization for the inoculated plants.

## 2. Materials and Methods

### 2.1. Experimental Design

An experiment with a factorial pot design was conducted in the greenhouses of Oregon State University. This experiment was a 6 × 2 × 3 factorial with inoculum type, biochar, and plant species, with 5 replications. Therefore, 180 pots were used in this experiment. To better understand the inoculum effects, we created a control or “sham” inoculum for each AMF source that was composed of the sterilized inoculum media plus a microbial wash from such inoculum source [56]. This control “sham” inoculum was made to control additional soil organisms except AMF. For the complete list of treatment combinations, see Table 1.

### 2.2. Seed Source

Seeds of all three species were field-collected. *P. spicata* was collected in the John Day Fossil Beds National Monument Clarno Unit during the summer of 2017. Seeds of *T. caput-medusae* were collected from the Crooked River National Grassland during the fall of 2018. Seeds of *V. dubia* were collected in the Phillip W. Schneider Wildlife Management area during the fall of 2019. Seeds of all species remained refrigerated to prevent loss of viability. Seeds of all target species were pre-germinated using a Hoffman walk-in germinator (Albany, OR, USA) in plastic containers using distilled water. One germinated seed was planted in each pot (Poly-tainer™ cans) of 2.4 L (0.65 gal), 15.2 cm (6 in) diameter and 17 cm (7 in) deep.

The soil used for this experiment was collected from a disturbed area in the Crooked River National Grasslands, a typical sagebrush grassland ecosystem where the three species of this study grow. The plant community of this ecosystem includes patches of *Artemisia tridentata* Nutt ssp. *tridentata*, *Poa bulbosa* L., *Taeniatherum caput-medusae* (L.) Nevski, and *Elymus elymoides* (Raf.) Swezey. Coordinates of the area from which the soil was sampled within the grasslands are 44°27′50″ N–121°0′57″ W. The soil collected from this area is a Lamonta series with a loam texture (44% sand, 22% clay, and 34% silt), moderate fine and a medium subangular blocky structure, a Munsell dry color 10YR 4/3 and wet color 10YR 2/2, 2.68% organic matter, neutral (pH 6.8), and a bulk density of 1.15 g/cm^3^. Before using the soil for our experiment, it was autoclaved using a pressure autoclave chamber (Consolidated Sterilizer Systems, Model SR-24F, Boston, MA, USA) for 90 min at 121 °C to remove all living microorganisms including AMF.

### 2.3. Inoculum Source

The three sources of inoculum that we tested were soil from the disturbed site (Inoculum A), soil from an undisturbed site (Inoculum B), and a commercial AMF inoculum (Inoculum C). Inoculum A was the live soil collected from the Crooked River National Grasslands on areas with previous history of disturbance such as plant-cover removal, presence of invasive species, plowing, and overgrazing and where both *T. caput-medusae* and *V. dubia* (exotic annual species) commonly grow. Inoculum B was live field soil collected from an undisturbed area in which *P. spicata* (native perennial species) commonly grows. Soil was collected under the canopy of individuals of *P. spicata*. Coordinates of the site where the soil was collected are 43°56′39″ N–121°0′19″ W. Plant species of the site where the late seral soil was collected include *P. spicata*, *Juniperus occidentalis* Hook., *Grayia spinosa* (Hook.) Moq., *A. tridentata* ssp. *tridentata*, and *Hesperostipa comata* (Trin. and Rupr.) Barkworth. Both live soils (Inoculum A and Inoculum B) were collected from a depth of 0–40 cm in a single location within each sampling site to minimize disturbance. Soil was stored in plastic containers to facilitate transportation; containers were kept at room temperature until used. Soils were used within less than a week to avoid loss of AMF propagules viability. Finally, Inoculum C was the AM120 standard (Reforestation Technologies International, Gilroy, CA, USA). The label content of this inoculum was 100% *Rhizophagus intraradices*.

### 2.4. Seeding and Inoculation

For seeding, we added 2 kg of autoclaved soil to each pot and then a layer of 100 g of the corresponding inoculum (A, B and C), followed by another layer of 100 g of autoclaved soil. For the biochar treatments, 5% weight [57] of biochar (Oregon Biochar Solutions, OR, USA) made of mixed conifer logging slash was mixed with autoclaved soil, the pots were filled to the same volume represented by the 2 kg of autoclaved soil, and then we added the inoculum layer, followed with the last layer of soil. This method of layering inoculum was selected to guarantee that the plant roots would be exposed to a layer of dense AMF propagules. Biochar was mixed in the soil rather than applied to the surface to maximize contact with the rhizosphere. A germinated seed from each plant species was planted in the pot.

Sham inoculum was made by mixing a microbial wash from each inoculum source with the same autoclaved inoculum, to create an inoculum with the original microbial community except for AMF [58]. Next, 100 mL of microbial wash was added. Microbial wash was made by mixing 700 g from the substrate (Inocula A, B and C) with 4 L of water, stirring by hand for a minute, and then allowing it to settle for 30 min. Settled solution was poured through multiple sieve sizes to trap AMF spores: first, through a 45 μm sieve; second, through a 25 μm sieve three times; and then through a 20 μm sieve [59]. The final solution was brought to a final volume of 13 L [59].

Once all the plants were established in their respective treatments, the pots were maintained at field capacity by watering until they started to drain. The greenhouse temperature was set to 25 °C daytime and 15 °C nighttime, with a daytime period from 6 am to 6 pm. We also supplemented the plants with grow lights when natural light intensity was <75 μM using Ultra Sun 400 W grow lights on Sun System^®^ lamps (Hawthorne Gardening Company, Vancouver, WA, USA). Plants were grown for 6 months. After the growing period, plants were manually harvested, roots were carefully extracted from the soil and washed, and a subsample was stored in 20 mL vials containing a mixture of 50% ethanol, 5% acetic acid, and 45% water for colonization measurements. Shoots and remaining roots were cut and stored in paper bags. Roots used for colonization counts were cleared and stained with trypan blue. Colonization was measured using the gridline intersect method under a microscope [60]. Roots were considered colonized if they contained AMF hyphae, vesicles, or arbuscules within their root cortex.

### 2.5. Statistical Analysis

Data were analyzed with R (2021) with a factorial ANOVA. We measured plant biomass production (dry shoot weight, dry root weight, and total) and root colonization percentage. We first tested if the sham inocula differed from the inocula for each plant species on biomass production using ANOVA. This was done to confirm that sham inocula did not have an effect on the plant species. Then, we excluded all sham inoculum data and centered our biomass analysis on the effects from each inoculum type (A, B, and C) x biochar (presence/absence of biochar) and their interaction for each plant species. Post hoc analysis Tukey’s test was conducted to test for differences after conducting the ANOVA.

## 3. Results

### 3.1. Root Colonization

**Figure 1:**
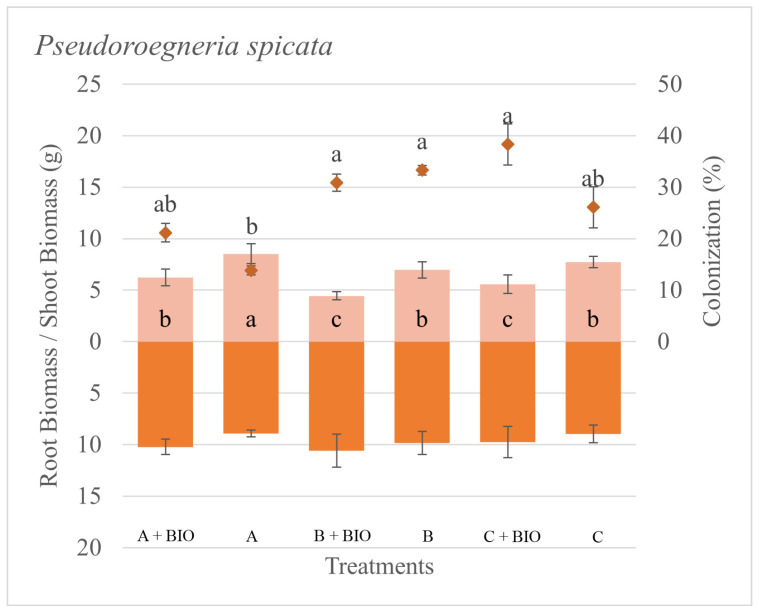
Shoot and root biomass (bars), root colonization (diamonds), and standard error of the mean (error bars) of *Pseudoroegneria spicata* grown with different sources of AMF inoculum (A, B, and C) and biochar (Bio). Different letters inside bars (shoot/root) and in squares (colonization) indicate significant differences among treatments (Tukey’s HSD).

**Figure 2:**
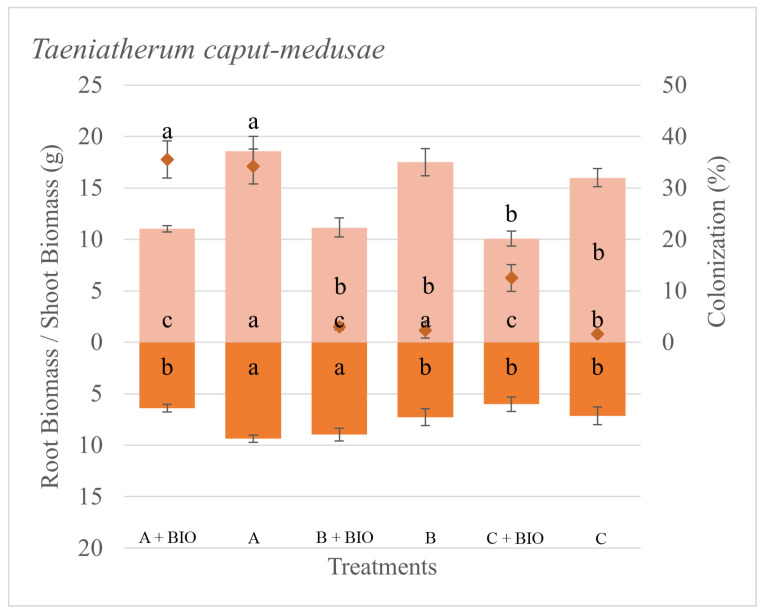
Shoot and root biomass (bars), root colonization (diamonds), and standard error of the mean (error bars) of *Taeniatherum caput-medusae* grown with different sources of AMF inoculum (A, B, and C) and biochar (Bio). Different letters inside bars (shoot/root) and in squares (colonization) indicate significant differences among treatments (Tukey’s HSD).

**Figure 3:**
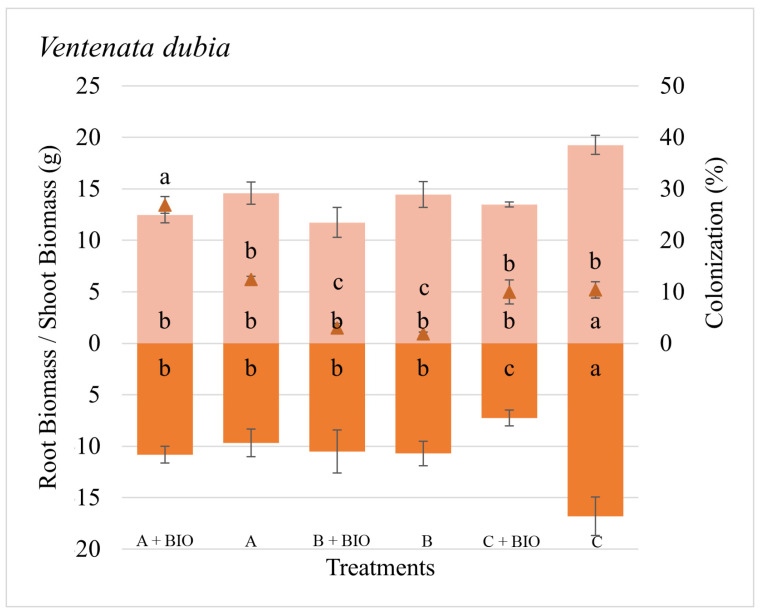
Shoot and root biomass (bars), root colonization (triangles), and standard error of the mean (error bars) of *Ventenata dubia* grown with different sources of AMF inoculum (A, B, and C) and biochar (Bio). Different letters inside bars (shoot/root) and in squares (colonization) indicate significant differences among treatments (Tukey’s HSD).

The differences in root colonization for each species were associated with the sources of inoculum and to a lesser extent with the biochar application (Table 2). The colonization was zero on all the sham controls. The root colonization for *P. spicata* was the lowest with Inoculum A, while all the other treatments resulted in a greater amount of colonization (Figure 1). The colonization of *T. caput-medusae* was greatest on both Inoculum A with and without biochar (Figure 2). The root colonization of *V. dubia* was greatest on Inoculum A with biochar compared to all the other treatments (Figure 3). These results suggest that each plant species had a different colonization response depending on the source of the AMF inoculum, and such a response can be associated with the plant’s seral status.

### 3.2. Biomass Production

The plant responses (biomass production) to AMF inoculation and biochar were dependent on the AMF source and presence/absence of biochar with positive, negative, and neutral biomass responses across the different inocula (Table 2). The total biomass of *P. spicata* was not particularly driven by either the different sources of inoculum or biochar application. *P. spicata* produced more root biomass than shoot biomass in all the treatments. However, the shoot biomass of *P. spicata* was greater when grown with Inoculum A without the biochar (Figure 1). The total, shoot, and root biomass of *T. caput-medusae* were greater when grown without biochar (Figure 2). The total and shoot biomass of *V. dubia* was found to be driven by biochar, the different inocula, and their interaction. For this species, the biomass was the greatest when grown with Inoculum C without biochar (Figure 3). We found that, in general, biochar did not increase biomass production for the evaluated species as we expected.

## 4. Discussion

### 4.1. Differential Colonization Responses

This study showed how three rangeland grass species (*P. spicata*, *T. caput-medusae*, and *V. dubia*) responded to three different sources of AMF inoculum: soil from an early seral (disturbed ecosystem), soil from a late seral (undisturbed ecosystem), and commercial AMF. Each inoculum type provided a different effect for the plant species, which is consistent with previous studies [13,61,62,63].

Highest levels of colonization for *P. spicata* were achieved with the commercial inoculum and with soil from the late seral ecosystem. This plant is a mycorrhizal species that gains competitivity when associated with AMF [51]. Interestingly, the levels of colonization of this species when inoculated in early seral soils are similar to those found in other studies (between 20% or lower) when grown in field soils [53]. Furthermore, we collected our undisturbed field soil below the canopy of already established *P. spicata* individuals to ensure the presence of an adequate AMF community [64]. Thus, for the restoration of *P. spicata* areas, the tested commercial inoculum or AMF extracted from a climax community might be useful.

The species *T. caput-medusae* and *V. dubia* were poorly colonized with AMF from late seral/undisturbed soils, and colonization was greater with early seral/disturbed soils and commercial AMF inocula with biochar. Those species were able to interact more with the commercial and early seral AMF community, possibly as early seral soils harbor fast-growing generalist AMF species [62]. Our evidence suggests that both annual species are colonized by AMF, but more research is needed to understand how they interact and respond to the AMF communities [61,62,63].

Biochar effectively influenced the mycorrhizal responses, and it increased colonization on *P. spicata* on early seral soils and commercial inocula, but this response was not observed on late seral soils. Therefore, our collected field soil could have been already conditioned for the growth of *P. spicata*, particularly as the invasives *T. caput-medusae* and *V. dubia* presented very low colonization levels when grown with this source and without biochar [62,65]. Biochar application on early seral soils did not increase colonization for *T. caput-medusae*, but it doubled colonization on *V. dubia*. Although not significant, the combination of biochar and commercial AMF apparently presented an increase in colonization for *T. caput-medusae*. This indicates a differential response and effect from each AMF source [30,32], and a possible differential effect from the biochar [66]. The reason for the colonization gains with biochar might be, as we hypothesize, an unexpected reduction in the soil nutrients as a result of the large surface area covered by the high biochar quantities used [43,49]. This reduction in nutrients might have triggered the plant response to associate with AMF.

Based on our results, both annual species responded as facultative mycorrhizal plants, as they were positively colonized by AMF, but the lack of colonization in some of the treatments did not prevent their growth [67,68]. Facultative mycorrhizal plants can keep low levels of colonization to gain benefits during stress periods or important growth stages [61], thus making them efficient even in low-nutrient degraded soils.

### 4.2. Biomass Production: Growth Responses

We found that both annual species produced more biomass compared to the perennial *P. spicata* and that biochar had little or even negative effects on the total biomass production of all species. While annual species are expected to produce more biomass in short periods of time [69], the apparent negative effects of biochar were unexpected as biochar is considered an amendment to favor plant growth [70]. Interestingly, under certain conditions biochar was found to produce negative growth responses [71]. We suspect that the high concentration of applied biochar may have influenced the negative growth response on the aboveground biomass by reducing the availability of nutrients such as nitrogen [43,46,49]. This needs further investigation, as we evenly mixed the biochar with the soil to maximize biochar contact with the rhizosphere and increase benefits [72]. However, this method might not be viable for large field-scale applications, and, thus, other methods of biochar application need to be further evaluated.

### 4.3. Differential AMF Sources on Sagebrush Steppe Plant Species

We found that each mycorrhizal inoculum produced different results in each plant species. We only sampled one late seral and one early seral ecosystem, so we cannot extrapolate our inocula effects to other ecosystems due to our limited soil sample size (i.e., one inoculum type as a single biological sample repeatedly assayed). Moreover, we only evaluated one perennial and two annual plant species; therefore, we cannot assume that these responses are dependent on a plant’s seral status. However, this research is important to understand the differential responses of rangeland plant species to contrasting inoculum sources. Furthermore, many questions remain unanswered about the invasive characteristics of our target species [11,73], while other research found that some invasive species can benefit from AMF [74] and biochar [46]. Therefore, each inoculum source needs to be carefully addressed and tested before application, as each plant species might have different requirements [63].

## 5. Conclusions

Based on our results, we accept both hypotheses: each mycorrhizal inoculum source had a differential response on each evaluated plant species, and, under certain conditions, biochar did increase colonization. Colonization was greater on *P. spicata* when inoculated with a late seral soil, which could indicate a long-term benefit for this perennial species and the presence of a mycorrhizal community conditioned for perennial species. On the other hand, the colonization of *T. caput-medusae* and *V. dubia* was greater when inoculated with early seral soils, which could favor their invasive status in disturbed areas. Our results are valuable for restoration efforts in rangelands dealing with invasive species. AMF inoculation and biochar application are easily applied, and previous research found some positive results; because of this, they are often recommended as viable options for restoration. However, based on our results, attention should be paid when applying biochar together with AMF in the field, as this could favor some invasive species and increase their growth. Therefore, we recommend a proper evaluation of each AMF inoculum source prior to designing a restoration program and continued assessment of the mycorrhizal responses of both native and invasive species.

## Figures and Tables

**Table 1:** Different combinations of 12 treatments of inoculum (Inoculum A: early seral soil; Inoculum B: late seral soil; Inoculum C: commercial AMF) vs. their respective sham-inoculant controls and biochar application (yes/no) used in the study for each of the 3 target species.

Treatment Combinations
Inoculum A, biochar
Inoculum A, no biochar
Inoculum A, sham, biochar
Inoculum A, sham, no biochar
Inoculum B, biochar
Inoculum B, no biochar
Inoculum B, sham, biochar
Inoculum B, sham, no biochar
Inoculum C, biochar
Inoculum C, no biochar
Inoculum C, sham, biochar
Inoculum C, sham, no biochar

**Table 2:** ANOVA results for total biomass, shoot, root, and colonization from *Pseudoroegneria spicata*, *Taeniatherum caput-medusae*, and *Ventenata dubia* grown for 6 months with different sources of mycorrhizal inoculum. Bold numbers indicate statistically significant results (*p* < 0.05).

Total Biomass	*P. spicata*		*T. caput-medusae*		*V. dubia*	
	*p*	F	*p*	F	*p*	F
Inoculum source	0.99	**0.001**	0.06	3.04	**0.03**	4.06
Biochar (+/−)	0.60	0.28	**<0.001**	47.59	**<0.001**	17.12
Interaction	0.65	0.43	0.18	1.81	**<0.001**	8.48
Shoot						
	*p*	F	*p*	F	*p*	F
Inoculum source	0.11	2.41	2.20	1.68	**0.007**	6.03
Biochar (+/−)	**<0.001**	13.8	**<0.001**	65.57	**<0.001**	17.71
Interaction	0.96	**0.03**	0.70	0.36	0.18	1.82
Root						
	*p*	F	*p*	F	*p*	F
Inoculum source	0.35	1.09	0.07	2.96	0.86	0.86
Biochar (+/−)	0.08	3.12	**0.05**	4.06	**0.02**	5.98
Interaction	0.43	0.85	**0.02**	4.40	**0.001**	8.27
Colonization						
	*p*	F	*p*	F	*p*	F
Inoculum source	**<0.001**	17.59	**<0.001**	51.70	**<0.001**	74.44
Biochar (+/−)	**0.02**	6.001	0.13	2.36	**<0.001**	18.98
Interaction	**0.05**	3.39	0.27	1.37	**<0.001**	16.72

## Data Availability

Not applicable.
